# How the Heart Was Involved in COVID-19 during the First Pandemic Phase: A Review

**DOI:** 10.3390/epidemiologia2010011

**Published:** 2021-03-22

**Authors:** Andrea Canalella, Ermanno Vitale, Francesca Vella, Paola Senia, Emanuele Cannizzaro, Caterina Ledda, Venerando Rapisarda

**Affiliations:** 1 Occupational Medicine, Department of Clinical and Experimental Medicine, University of Catania, 95123 Catania, Italy; ancanalella@gmail.com (A.C.); fracescav.89@libero.it (F.V.); paosenia@hotmail.it (P.S.); cledda@unict.it (C.L.); 2 Department of Sciences for Health Promotion and Mother and Child Care “Giuseppe D’Alessandro”, University of Palermo, 90127 Palermo, Italy; emanuele.cannizzaro@unipa.it

**Keywords:** Coronavirus, Sars-CoV-2, COVID-19, cardiovascular disease, cytokine storm

## Abstract

Coronavirus disease (COVID-19) was first observed in Wuhan, Hubei Province (China) in December 2019, resulting in an acute respiratory syndrome. Only later was COVID-19 considered a public health emergency of international concern and, on 11 March 2020, the WHO classified it as pandemic. Despite being a respiratory virus, the clinical manifestations are also characterized by cardiological involvement, especially in patients suffering from previous comorbidities such as hypertension and diabetes mellitus, its complications being potentially serious or fatal. Despite the efforts made by the scientific community to identify pathophysiological mechanisms, they still remain unclear. A fundamental role is played by the angiotensin 2 converting enzyme, known for its effects at the cardiovascular level and for its involvement in COVID-19 pathogenesis. The goal of this paper was to highlight the mechanisms and knowledge related to cardiovascular involvement during the first pandemic phase, as well as to emphasize the main cardiological complications in infected patients.

## 1. Introduction

Since the outbreak of severe acute respiratory syndrome (SARS) 18 years ago, a large number of SARS-related coronaviruses (SARSr-CoVs) have been discovered in their natural reservoir host, bats [1,2,3,4]. Previous studies have shown that some bat-resident SARSr-CoVs may potentially infect humans [5,6,7]. On 12 December 2019, a new coronavirus (2019-nCoV), caused an epidemic of acute respiratory syndrome in humans in Wuhan, China [8]. On 31 January 2020, the World Health Organization (WHO) categorized COVID-19 a public health emergency worldwide, and on 11 March 2020, it was finally characterized as a pandemic [9]. As of 10 May 2020, more than 4 million COVID-19 cases and 280,000 deaths have been reported globally, reflecting the increased infectivity and severity of this virus; however, the lack of widespread testing availability means figures are probably even higher than those reported [10]. The SARS-CoV-2 is a β-coronavirus, an enveloped, non-segmented positive-sense RNA virus (subgenus sarbe-covirus, Orthocoronavirinae subfamily) [11]. Coronaviruses (CoVs) are divided into four genera, including α−/β−/γ−/δ-CoV. α- and β-CoV can infect mammals, while γ- and δ-CoV tend to infect birds [12]. The SARS-CoV-2 receptor association domain (RBD) is optimized for association with human angiotensin converting enzyme 2 (ACE2) with an efficient solution different from those envisaged at first [13,14]. The high-affinity link of SARS-CoV-2 spike protein to human ACE2 is most probably the result of natural selection on a human or human-like ACE2 that allows another optimal binding solution to arise [15]. The estimated incubation period of the novel CoV ranges from 2 to 14 days. However, some cases had an incubation period of 21, 24, or 27 days [16]. The clinical characteristics of mild COVID-19 seem to include symptoms common to other viral infections (i.e., cough, fever, myalgias, fatigue, and diarrhea) as well as laboratory abnormalities such as lymphopenia [17], although knowledge of the clinical nature of the disease is evolving daily [18,19]. In severe cases, COVID-19 may present itself as pneumonia, which can develop into acute respiratory distress syndrome (ARDS), with or without cardiogenic shock, to which elderly populations with pre-existing medical comorbidities are most vulnerable [18,19,20,21]. Most current reports on COVID-19 have described the clinical manifestations of cardiovascular disease (CVD) in these patients. Given the global importance of the disease and the remarkably adverse prognostic impact of cardiac involvement, further research is needed to comprehend incidence, mechanisms, clinical presentation, and outcomes of various CVD manifestations in COVID-19 subjects [22]. Even though the respiratory tract is the primary target of SARS-CoV-2, the cardiovascular (CV) system may get involved in various ways [22]. CVD was a common comorbidity in patients with COVID-19 predecessors, SARS and Middle East respiratory syndrome (MERS) [23]. In SARS, the prevalence of diabetes mellitus (DM) and CVD was 11% and 8%, respectively, and the presence of either comorbidity increased, 12 times, the risk of death [24,25]. DM and hypertension were prevalent in ≈50% of patients with MERS; CVD was present in ≈30% of patients with MERS infection [26].

The aim of this study was to analyze CV involvement/complications following SARS-CoV-2 infection during the first pandemic phase.

## 2. Materials and Methods

This review was carried out in line with the PRISMA statement (Preferred Reporting Items for Systematic reviews and Meta-Analyses).

### 2.1. Literature Search

SCOPUS and Medline (using PubMed as the search engine) databases were searched in order to recognize relevant research available until 23 of June to examine CV complications associated with SARS-COV-2 infection.

MeSH entry terms used were “SARS-CoV-2” AND “cardiovascular disease”; “SARS-CoV-2”; “COVID-19”.

A search of the research articles that were suitable for inclusion in this review was also done, and the research-significant papers were therein collected and reviewed.

### 2.2. Inclusion and Exclusion Criteria

The following inclusion criteria were adopted: studies that assessed the SARS-CoV-2 in association with CVD. The following exclusion criteria were applied: scientific articles that were not published in English and/or conference abstracts, and/or reviews or letters.

For duplicate studies, only the article with further detailed information was included.

### 2.3. Quality Assessment and Data Extraction

Two reviewers (A.C. and E.V.) evaluated articles separately. The title, abstract, and full text of each potentially relevant study were reviewed. Any divergence on the eligibility of the studies was ascertained through debate or by consulting an additional reviewer (C.L.). The following information was extracted from all qualified papers: authors, year of publication, nationality of subjects, and study characteristics.

## 3. Results

### 3.1. Characteristics of Eligible Studies

**Figure 1:**
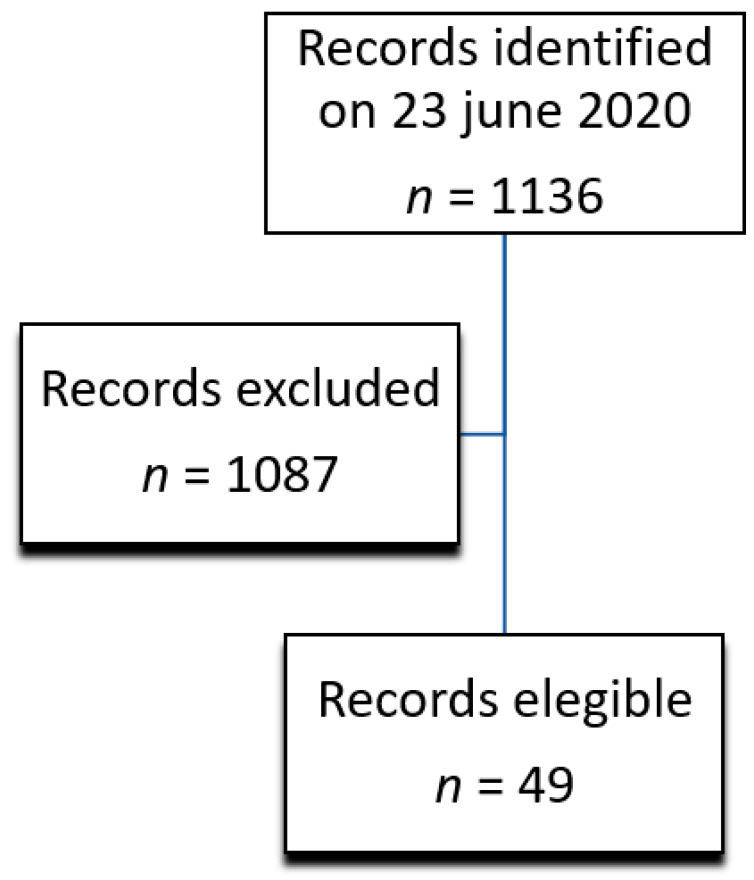
Flow diagram illustrating in/excluded studies in this review.

After a free search for scientific literature by reviewers, a total of 1136 (100%) documents were collected. Of these, 230 (20%) were ruled out because of their reviews, 746 (65%) were only abstracts, 34 (3%) were disqualified after subsequent analysis of the title, and 77 (7%) were ruled out because they were not in English. In conclusion, 49 (4%) studies satisfied the inclusion criteria and were encompassed in the systematic review. Figure 1 represented a flowchart descriptive of the choice of the articles.

Before June 2020 there were few studies defining the pathophysiological nature of COVID-19 and there were substantial uncertainties about CV-involvement complications.

From the analysis of the 49 studies, the involvement of the CV apparatus in COVID-19 infection was described through the following points: (a) COVID-19 and myocardial injury, (b) acute myocardial infarction (AMI), (c) cardiac arrhythmia and cardiac arrest, (d) heart failure (HF) and cardiogenic and mixed shock, (e) venous thromboembolism event (VTE), (f) heart transplantation, and (g) long-term sequelae of SARS-CoV-2 infection.

Table 1 summarizes details of the included research articles. From the analysis of the 49 eligible studies it was observed that the total number of subjects analyzed was 11,566. Of these subjects, 64% (*n* = 7386) were males and the mean age was 63.1 ± 8.8 years. The average number of hospitalization days was 28.4 ± 17.3. The percentage of patients with hypertension (Hp) was 66% and with diabetes mellitus (DM) it was 32.5%. The percentage of patients needing ICU was 1.2%; 47.6% of the whole sample deceased, while 52.4% survived.

The most frequent complications observed were acute cardiac injury (ACI) (*n* = 767) [20,29,31,56,58,62,63,64,65,68,70,74], cardiogenic shock (*n* = 220) [20,44,58,64,68], 41 Hp (*n* = 41) [27,45], HF (*n* = 159) [29,31,37,42,67,74], cardiovascular disease (CDV) (*n* = 70) [27,34,52], arrhythmia (arrh) (*n* = 45) [30,31,55,67], acute coronary syndrome (ACS) (*n* = 32) [30,45,60,61], disseminated intravascular coagulation (DIC) (*n* = 27) [30,74], cardiac arrest (*n* = 21) [30,47,54], venous thrombo-embolism (VTE) (*n* = 12) [74], and acute myocardial infarction (AMI) (*n* = 8) [43,53,60,67].

### 3.2. Covid-19 and Myocardial Injury

Severe myocardial damage was the most commonly described CV complication in COVID-19.

Any of the mechanisms described above can bring to acute cardiac injury (ACI) and rise in cardiac troponins in COVID-19 patients. The relative role of these diverse mechanisms has not been described but direct (i.e., non-coronary) myocardial injury due to viral myocarditis or the effect of systemic inflammation seems to be the most common mechanism. These observations were sustained by a previous autopsy study of patients who had died due to SARS during the Toronto SARS outbreak [75]. Oudit et al. [75] detected viral ribonucleic acid in 35% of the autopsied human heart samples, providing evidence for direct myocardial damage by the virus.

A report from China’s National Health Commission showed that almost 12% of patients without known CVD had elevated troponin levels or cardiac arrest during hospitalization [28].

Early reports indicate that there are two patterns of COVID-19 myocardial damage [23]. One study proved that at 4 days after symptom onset, median high sensitivity troponin (hs-cTnI) levels were 8.8 pg/mL in non-survivors vs. 2.5 pg/mL in survivors. During follow-up, the median hs-cTnI among survivors did not change significantly (2.5 to 4.4 pg/mL); whereas it rose to 24.7 pg/mL on day 7, to 55.7 pg/mL on day 13, to 134.5 pg/mL on day 19, and to 290.6 pg/mL on day 22 in non-survivors [29]. In particular, the median time to death from symptom onset was 18.5 days (interquartile range, 15 to 20 days) [23].

A murine model proved pulmonary infection with SARS-CoV also precipitated an ACE2-dependent myocardial infection [75]. Other suggested mechanisms of COVID-19–related cardiac involvement include a cytokine storm, mediated by an excessive response among subtypes of T helper cells [29], and hypoxia-induced excessive intracellular calcium leading to heart myocyte apoptosis [28].

Collateral tissue injury and the inflammatory processes that follow vasodilation, endothelial permeability, and leukocyte recruitment lead to further pulmonary damage, hypoxemia, and CV stress. In a subset of patients, the host inflammatory response continues to amplify (even with diminishing viral loads) and results in systemic inflammation [19,76].

This systemic toxicity, in turn, has the potential to injure distant organs [77]. Reports of myocarditis in COVID-19 without any trace of direct viral infiltration implicate the heart as one such target of systemic inflammation [78]. Exaggerated systemic inflammation, or cytokine storm, may correlate with lymphocytopenia and is a hallmark of acute disease [79]. Systemic inflammation represents an advanced stage of the acute illness, characterized by multiple organ failure (MOF) and elevation of key inflammatory markers [80]. Based on clinical data, these inflammatory markers include interleukin (IL)-6, IL-2, IL-7, tumor necrosis factor (TNF-α), interferon (IFN-γ), inducible protein (IP-10), monocyte chemoattractant protein (MCP-1), macrophage inflammatory protein (MIP-1α), granulocyte-colony stimulating factor (G-CSF), C-reactive protein (CRP), procalcitonin, and ferritin [20,29,79,81,82]. Biomarkers of heart injury and electrocardiographic abnormalities correlate with elevated inflammatory markers [38,83]. Autopsies show inflammatory infiltrates composed of macrophages and, to a lesser extent, CD4 + T cells [78,84]. These mononuclear infiltrates are associated with regions of cardiomyocyte necrosis, which, by Dallas Criteria, defines myocarditis [18,85]. So far, however, there are no data showing evidence of SARS-CoV-2 within myocardial tissue. Postmortem real-time polymerase chain reaction (rt-PCR) analyses of cardiac tissue from the SARS epidemic, detected the viral genome in 35% (*n* = 7/20) of patients who died from SARS [75]. Of note, these hearts also had reduced levels of ACE2 and increased hypertrophy [75,86]. It remains unclear how much of the heart damage was attributable to direct viral infection versus indirect systemic toxicity. Furthermore, it is unclear which cell populations within the myocardium are most vulnerable to infection and systemic inflammation. Myocardial pericytes, which play an important role in maintaining endothelial function, express ACE2 abundantly [86]. Dysfunction in cardiac pericytes and endothelial cells, either due to direct infection or global inflammation, can lead to disruption in the coronary microcirculation with downstream ischemic consequences, but the relationship to COVID-19 is purely conjectural [77]. In June 2020, there was not enough information to determine whether myocarditis in COVID-19 more commonly causes cardiac failure with preserved ejection fraction or reduced ejection fraction, although most patients with uncomplicated lymphocytic myocarditis were admitted with normal heart function [73,87,88,89]. Consistent with the possibility that cardiac failure with preserved ejection fraction (EF) may be more common, a case report from Wuhan highlights the coexistence of elevated TnI and brain natriuretic peptide (BNP) in a critically ill COVID-19 patient with an echocardiographic (ECO) EF of 60% [90]. ECO evaluation is more likely to show a focal wall motion abnormality with active, significant acute coronary syndrome (ACS) while severe forms of COVID-19-related myocarditis will show either no wall motion flaws or global wall motion dysfunction [17,21]. Electrocardiography (ECG) and ECO abnormalities in COVID-19 setting are markers of illness severity and are associated with worse outcomes [17,38,51].

### 3.3. Acute Myocardial Infarction (AMI)

Acute systemic inflammation enhances the risk of atherosclerotic plaque disruption and AMI [21,38,74,91,92]. Due to extensive inflammation and hypercoagulability, the risk of AMI is likely present in COVID-19 patients [17,21]. The treatment of AMI is controversial in these subjects. In patients diagnosed with an ST elevation myocardial infarction (STEMI) and COVID-19, the American College of Cardiology (ACC) maintains that while fibrinolysis may be considered in those with “low risk STEMI”—defined by inferior STEMI with no right ventricular involvement or lateral AMI without hemodynamic compromise—percutaneous coronary intervention (PCI) is more commonly carried out in most institutions and remains the elective surgery [21]. If PCI is chosen, medical staff ought to wear appropriate personal protective equipment (PPE) and a full decontamination of the catheterization laboratory should be performed following the procedure. For suspected COVID-19 in an Non-ST-segment elevation myocardial infarction. (NSTEMI) setting, diagnostic testing is recommended prior to catheterization; the ACC claim that, in adequately selected, confirmed COVID-19 patients, conservative therapy may be sufficient [93]. Patients who are hemodynamically unstable in the setting of NSTEMI should be treated similarly to those with STEMI [21].

Analysis by Kwong et al. [74] proved that patients with severe respiratory infections were at elevated risk to further developing AMI after influenza (incidence ratio: 6.1; 95% CI: 3.9 to 9.5) and after non-influenza viral illnesses including other CoVs species (incidence ratio: 2.8; 95% CI: 1.2 to 6.2).

### 3.4. Cardiac Arrhythmia and Cardiac Arrest

Cardiac arrhythmias are another typical CV phenomenon described in patients infected with COVID-19. Though nonspecific, heart palpitations were part of the incoming symptomology in 7.3% of patients in a cohort of 137 patients admitted for COVID-19 [94].

In hospitalized COVID-19 subjects, cardiac arrhythmia was detected in 16.7% in a Chinese cohort of 138 patients and was more common in ICU patients than in non-ICU ones (44.4% vs. 6.9%) [82]. However, new onset of malignant tachyarrhythmias in the setting of troponin elevation should arouse suspicion of underlying myocarditis [95,96].

### 3.5. Heart Failure (HF) and Cardiogenic and Mixed Shock

In June 2020 it was unclear whether HF was more commonly due to exacerbation of pre-existing left ventricular dysfunction or to new cardiomyopathy (either due to myocarditis or stress cardiomyopathy) [97]. Right HF and associated pulmonary hypertension should be also taken into account, particularly in events like severe parenchymal lung disease and ARDS. Zhou et al. [29] reported that heart failure was observed in 23.0% of patients with COVID-19 presentations. Notably, HF was more commonly detected than acute kidney injury in this cohort and was more common in patients who did not survive hospitalization than in those who did (51.9% vs. 11.7%) [29]. The chief clinical symptom of COVID-19 is acute respiratory illness, which may lead to ARDS manifested as ground-glass opacities on chest imaging [98] and hypoxemia. However, similar features may be seen in the case of de-novo or coexisting cardiogenic lung edema. As such, it is important to consider cardiogenic or mixed cardiac plus primary pulmonary causes of respiratory manifestations in COVID-19. Historically, right heart catheterization has been used to assess pulmonary capillary wedge pressure to aid in this distinction, although this has been removed from the Berlin criteria used for diagnosing ARDS. Rather, the Berlin criteria use timing of symptom onset, imaging with bilateral pulmonary opacities, and lack of volume overload to identify patients with ARDS [99]. In many cases, serum brain natriuretic peptide and echocardiography can help clarify the diagnosis [100,101]. However, if these tests are unclear and there remains concern for mixed presentation, pulmonary artery catheterization ought to be taken into account in select cases to assess filling pressures and cardiac output and to guide clinical decision making, given the different management approaches for ARDS and cardiogenic shock. Further studies regarding the effectiveness of extra corporeal membrane oxygenation (ECMO) support in advanced COVID-19 are warranted, including which patients may (or may not) benefit and whether associated left ventricular draining should be done [102].

### 3.6. Venous Thromboembolism Event (VTE)

COVID-19 patients also have increased risks of VTE [103,104]. Systemic inflammation, abnormal coagulation status, multiorgan dysfunction, and critical illness are all potential contributing factors to the increased risk of VTE [20,29,103,104,105]. Studies suggest substantial coagulation pathway abnormalities in patients with COVID-19, including elevated D-dimer [20,29,103,104,105]. A study of 25 patients with COVID-19 pneumonia revealed that an elevated D-dimer was present in all subjects with a median of 6.06 micrograms/mL, with 10 patients having pulmonary embolism (PE) diagnosed on computed tomographic pulmonary angiography (CTPA) [106]. Patients with confirmed PE on CTPA showed a median D-dimer level of 11.07 micrograms/mL [106]. D-dimer levels greater than 1 μg/mL were associated with an increased risk of death during hospitalization (odds ratio 18.4) in COVID-19-infected patients [29]. One study suggested that anticoagulation, mainly with low molecular weight heparin, may be associated with reduced mortality in severe COVID-19 infections or those with D-dimer greater than six times as the upper normal limit [107]. Similarly, microthrombi in the segmental pulmonary arteries have been observed on autopsy [108]. However, these findings could be secondary to cellular debris rather than microthrombi and, in some cases, they could be secondary to disseminated intravascular coagulation (DIC), as seen with sepsis from other etiologies [108]. Coagulation factors Xa and IIa have been shown capable of cleaving the SARS-CoV-2 spike protein and may, hence, promote infectivity [109]. Therefore, anticoagulation might inhibit this process and prevent SARS-CoV-2 replication.

### 3.7. Heart Transplantation

Apart from the mechanisms by which COVID-19 can affect patients with CVD risk factors, it is also important to consider COVID-19 in the context of especially fragile patients, such as those individuals awaiting transplantation or those who have already undergone transplantation [17]. There are now case reports of COVID-19 infection among heart transplant patients; formal treatment guidelines in these patients currently do not exist [17]. Heart allocation teams need to consider the optimal screening strategies to prevent severe infection in recipients including whether all donor hearts should be screened, given the existence of asymptomatic COVID-19, versus limiting screening to patients with a history of symptoms or exposure of COVID-19 [110,111]. Similarly, it is advisable to consider screening recipients for a history of symptoms or exposure of COVID-19 to avoid a post-transplant flare. Utmost infection control measures must be taken when interacting with these vulnerable immunosuppressed patients.

### 3.8. Long-Term Sequelae of SARS-CoV-2 Infection

**Figure 2:**
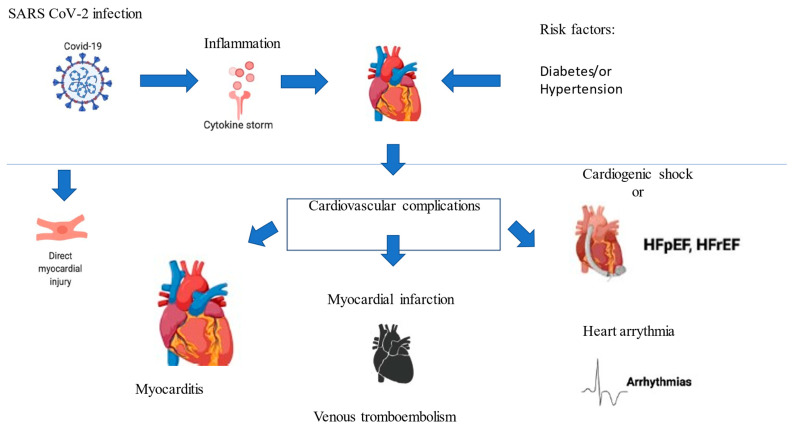
CV complications during COVID-19 infection [75].

CV complications are likely to occur even after recovery from illness. COVID-19 is a nascent pandemic and, therefore, long-term sequelae are unknown, but there are reports of complications occurring soon after major severe symptoms have disappeared. Figure 2 shows a graphic summary of what may happen to the heart after SARS-CoV-2 infection. A case report from Italy describes fulminant myocarditis in a convalescent patient one week after her respiratory symptoms resolved [73]. This suggests that background inflammation can go on and evolve silently, manifesting later in an insidious way. Even after apparently complete recovery, however, there may be chronic sequelae [77]. The previous SARS epidemic has been informative because sufficient time has elapsed for long-term follow-ups. A substantial proportion of survivors from the epidemic developed pulmonary fibrosis, avascular necrosis, and dyslipidemia [112,113]. The latter manifestations are chiefly important as they represent CV risk factors. In addition, hospitalization for pneumonia has been linked to increased short- and long-term risks for CVD, and this is especially true if there are heart complications at the index hospitalization [91,114]. Thus, cardiac involvement may persist long after resolution of the severe illness. Wang et al. [115] observed that 66 out of 70 hospitalized patients were showing some amount of lung damage on CT scans, and more than half might be the type of patient for whom this develops into scars. Meng et al. [116] suggest that this is not just for critically ill patients; and showed that out of 58 asymptomatic patients, 95 percent had also revealed opacities of the frosted glass in their lungs. More than 25% of these individuals continued to experience symptoms within a few days [116]. Melina et al. [117] declared that these kinds of tissue changes can cause permanent damage and the pulmonary function never recovers; their ability to perform normal activities never returns to baseline [117]. Further studies are needed to clarify the prognostic implications of SARS-CoV-2 infection.

## 4. Discussion

COVID-19, caused by SARS-CoV-2, is a global pandemic that has affected hundreds of thousands of people and is evolving in real time. The ACE2 enzyme plays a crucial role in CV and immune systems involved in cardiac function, and in the development of hypertension and diabetes mellitus it has been identified as a functional receptor for coronaviruses, including SARS-CoV and the new SARS-CoV-2 [118]. SARS-CoV-2 infection is triggered by the binding of the virus surface protein, called “spike”, to ACE2 [118]. Other proposed mechanisms of myocardial injury include a cytokine storm triggered by an imbalanced response from type 1 and type 2 T helper cells, and respiratory dysfunction and hypoxemia caused by COVID-19, resulting in damage of myocardial cells. The massive inflammatory response combined with hemodynamic changes associated with severe disease can carry the risk of atherosclerotic plaque rupture in sensitive patients, enhancing the risk of AMI and the abnormal state of coagulation and multi-organ dysfunction, considered potential factors contributing to the increased risk of VTE. There is a proclivity for SARS–CoV-2 binding to endothelial cells with resulting inflammation [119]. Kawasaki-like disease with accompanying toxic shock syndrome or multi-systemic inflammatory disease has been reported in children with COVID-19 [119].

The SARS-CoV-2 mainly invades alveolar epithelial cells, causing respiratory symptoms that are more acute in CVD patients. CV comorbidities are common in COVID-19 patients who turn out to be at higher risk of morbidity and mortality. It is not known whether the presence of CV comorbidity conditions poses independent risks or if these are conveyed by other factors (e.g., age). Myocardial injury is present in >25% of critical cases and appears in two patterns—severe myocardial injury and dysfunction on presentation and myocardial damage that develops as illness severity intensifies. High prevalence of arrhythmia might be, in part, attributable to metabolic disarray, hypoxia, or neurohormonal or inflammatory stress in the setting of viral infection in patients with or without existing CVD. Right-heart failure and related pulmonary hypertension ought to be also taken into account, in particular when severe parenchymal lung disease and ARDS are at play.

Furthermore, many recent studies have demonstrated a large increase in Takotsubo Syndrome incidence associated to Covid-19 [120]. However, it remains uncertain whether heart failure is more commonly due to exacerbation of pre-existing left ventricular dysfunction than to novel cardiomyopathy (due to myocarditis or stress cardiomyopathy) [121]. As to CV patients, measures should be evaluated in order to bring the risks of COVID-19 transmission to patients and healthcare staff close to none. An important mechanism to help prevent transmission is the use of telemedicine. This technology, already used by many major healthcare systems worldwide, is ideal in public health crises as it allows patients to undergo triage by curtailing exposure of patients and healthcare staff to potential infection [17]. Additionally, telemedicine offers specialists an opportunity that might not otherwise be available for diagnosing patients. Although there are currently obstacles to implementing telemedicine globally, such as coordination of tests in high-risk patients, this is a technology that is likely to prove crucial for promoting viral containment [122]. Other essential guidelines are to reduce non-essential/non-urgent health care operator–patient interactions (e.g., social distance) as much as possible, and to limit elective cardiac catheterization and operating room and echocardiographic procedures. If such procedures are needed, the number of necessary staff should be kept to a minimum. In months to come, efforts towards evaluating new therapies will be crucial to the treatment of this virus, and as this process develops, further assessment of the intricate interplay among COVID-19, CVD, and the various stakeholders involved including patients, health care workers, and systems will be important in order to improve outcomes in at-risk and infected patients. Prospective randomized clinical trials and cohort studies are in progress and will be vital to help treat patients affected by this virus.

## 5. Conclusions

The results of this review show that the cardiac muscle, after the lung, is a critical organ in SARS-CoV-2 infection. This agent can affect it with direct infection or cause heart damage through a cytokine-mediated inflammatory process. In the latter, various symptoms may occur, ranging from cardiac arrhythmia to acute myocardial infarction up to cardiac arrest. The main weakness of this article was that this is a new pathogen and little is known about the mechanisms underlying COVID-19 pathogenesis, clinical manifestations, or complications.

Furthermore, not all long-term sequelae and how they can affect the health of individuals were known; for these reasons, cardiac examination is extremely important while assessing patients’ health. Indeed, the heart check must be of vital importance in re-admission after COVID-19 disease.

The strengths of the study were the limited period observed and the remarkable specificity and sensitivity of the diagnostic processes aimed at distinguishing between symptomatic and asymptomatic subjects, which were useful to reduce the infection.

Therefore, further studies will be needed to better understand the pathophysiological mechanisms underlying the heart involvement following COVID-19 infection.

## Figures and Tables

**Table 1:** Main results of eligible studies.

Reference	N. Subjects	Day/Hospital	Sex	Age (Years)	Hp	DM	ICU	Outcomes	Complications
Huang et al. [20]	41	4–8/Wuhan	30 ♂, 11 ♀	41.0–58.0	6	8	13	12 dec	5 ACI, 3 shock
Du et al. [27]	179	43/Wuhan	97♂82♀	57.6 ± 13.7	58	33	n.r.	21 dec, 158 sur	13 Hp; 12 CVD
Zheng et al. [28]	5	n.r./China	n.r.	n.r.	n.r.	n.r.	4	n.r.	n.r.
Zhou et al. [29]	191	8–14/Wuhan	119 ♂72 ♀	46.0–67.0	58	36	50	137 sur, 54 dec	44 HF; 37 coagulopathy, 33 ACI
Du et al. [30]	85	10.1 ±6.2/Wuhan	62 ♂23 ♀	65.8 ± 14.2	32	19	n.r.	85 dec	7 cardiac arrest, 4 ACS, 2 arrh, 1 DIC
Wang et al. [31]	339	15–28/Wuhan	173 ♀166 ♂	65–76	138	54	n.r.	274 sur, 65 dec	70 ACI,35 arrh, 58 HF
Feng et al. [32]	476	12–24/China	271 ♂205 ♀	40–64	113	49	n.r.	403 sur, 23 dec	n.r.
Li et al. [33]	83	5–12/China	34 ♂49 ♀	32–62	33	n.r.	n.r.	n.r.	n.r.
Yan et al. [34]	193	7–16/Wuhan	114 ♂79 ♀	49–73	73	48	32	108 dec, 85 sur	31 CVD
Li et al. [35]	225	25/Hanchuan (China)	120 ♂105 ♀	50 ± 14	20.89%	n.r.	n.r.	2 dec, 223 sur	n.r.
Liang et al. [36]	1590	n.r./China	904 ♂674♀	48.9 ± 16.3	807	390	198	297 dec	n.r.
Gao M et al. [37]	35	14/Guangdong (China)	20 ♂15 ♀	50.0–84.0	9	n.r.	n.r.	2 dec	2 HF
Guo et al. [38]	187	26/Wuhan	91 ♂	58.50	61	28	n.r.	43 dec	11 ventricular fibrillation/ventricular tachycardia
Deng et al. [39]	112	22–38/Wuhan	57 ♂55 ♀	49.0–70.8	36	19	26	14 dec	n.r.
Itelman et al. [40]	162	38/Israel	105 ♂	52 ± 20	49	30	24	5 dec	n.r.
Gao et al. [41]	54	1–15/China	24 ♂30 ♀	60.4 ± 16.1	12	8	n.r.	18 dec	n.r.
Dong et al. [42]	4	68/Wuhan	4 ♂	11–67	1	1	2	2 dec, 2 sur	4 HF
Menter et al. [43]	21	n.r./Switzerland	17 ♂4 ♀	53–96	21	7	n.r.	21 dec	1 AMI, 3 peracute myocardial cell necrosis,
Qin et al. [44]	1875	8–18/Wuhan	945 ♂ 930 ♀	51–70	641	295	n.r.	159 dec	84 shock
Zhang et al. [45]	541	n.r./Wuhan	255 ♂	69.66 ± 10.94	125	62	n.r.	53 dec	28 hp, 17 ACS, 4 abnormal heart rhythms
Gao et al. [46]	2027	30–50/Wuhan	1027 ♂	55.38 64.24	850	387	n.r.	56 dec	n.r.
Shao et al. [47]	136	4–11/Wuhan	46 ♀90 ♂	61–77	41	27	23	n.r.	10 cardiac arrest
Pan et al. [48]	124	11–27/China	85 ♂39 ♀	61–75	62	25	91	89 dec, 35 sur	n.r.
Chen et al. [49]	54	32/China	36 ♂	44.9–70.3	16	15	n.r.	n.r.	3 ventricular tachycardia, 3 HF
Xie et al. [50]	140	6–26/Wuhan	72 ♂68 ♀	47–68	40	20	n.r.	36 dec	n.r.
Shi et al. [51]	416	1–30/Wuhan	211 ♀	21–95	127	60	n.r.	57 dec	n.r.
Li p et al. [52]	204	7–14/Wuhan	104 ♀	60–95	74	36	n.r.	76 dec	27 CVD
Edler et al. [53]	80	n.r./Germany	46 ♂34 ♀	52–96	26	19	17	80 dec	2 AMI
Creel-Bulos et al. [54]	5	12/n.r.	3 ♂2 ♀	42–76	3	2	5	3 dec, 2 sur	4 cardiac arrest
Ferguson et al. [55]	72	4–13/N.Carolina (USA)	38 ♂34 ♀	43.4–70.6	26	20	21	6 dec	6 arrh
Yang et al. [56]	200	2−7/Hubei (China)	98 ♂102 ♀	55	45	21	29	15 dec	20 ACI
Ren et al. [57]	87	n.r./Wuhan	63 ♂24 ♀	51 ± 12	28	20	n.r.	n.r.	n.r.
Zhao et al. [58]	1000	7–14/Wuhan	466 ♂534 ♀	46.70	282	118	63	119 dec	81 shock, 116 ACI
Goicoechea et al. [59]	36	0–15/Spain	23 ♂	71 ± 12	35	23	n.r.	25 sur, 11 dec	n.r.
Lodigiani et al. [60]	388	14–24/Milan (Italy)	264 ♂	55–75	183	88	61	130 dec	4 ACS 4 AMI
Xie et al. [61]	62	30/China	35 ♀	53.3–73.0	24	13	n.r.	n.r.	7 ACS
Buckner et al. [62]	105	24/Seattle (USA)	53 ♂52 ♀	69	62	35	34	35 dec	13 ACI
Zheng et al. [63]	34	49/China	23 ♂11♀	66	22	8	34	0 dec	13 ACI
Wan et al. [64]	135	3–10/China	72 ♂63 ♀	36–55	13	12	n.r.	1 dec	10 ACI, 1 shock
Rath et al. [65]	123	10.2 ± 7.5/Germany	77 ♂	68	86	30	56	107 sur, 16 dec	6 ACI
Biagi et al. [66]	1050	0–41/Piacenza (Italy)	230 ♂	40–98.5	235	72	320	320 dec	n.r.
Sabatino et al. [67]	76	44/Italy	36 ♀	34.7	5	1	1	0 dec	2 arrh, 5 HF, 1 AMI, 1 pericardial effusion
Shi et al. [68]	306	15/Wuhan	156 ♀150 ♂	56.0–72.0	131	153	17.6%	47 dec, 259 sur	73 ACI, 48 shock
Galloway et al. [69]	1157	2–28/London (U.K.)	666 ♂	71	611	408	157	244 dec	n.r.
Palmieri et al. [70]	3032	11/Italy	n.r.	368 < 65 y 2.664 ≥ 65 y	2071	914	n.r.	3032 dec	314 ACI
Wei et al. [71]	101	7/Sichuan (China)	54 ♂	49	21	14	31	3 dec	n.r.
Jain et al. [72]	459	14/Connecticut (USA)	64 ♂	68.2 ± 15.2	61	50	60	3 dec	n.r.
Inciardi et al. [73]	99	11.4 ± 6.5/Brescia (Italy)	80 ♂19 ♀	67 ± 12	63	30	12	26 dec	12 VTE, 3 arterial thrombo-embolism
Chen et al. [74]	274	6–17/China	171 ♂103 ♀	44.0–70.0	93	47	n.r.	161 sur, 113 dec	89 ACI, 43 HF, 21 DIC

n.r., not reported; HF, heart failure; ACS, acute coronary syndrome; ACI, acute cardiac injury; DIC, disseminated intravascular coagulation; CVD, cardiovascular disease; VTE, venous thrombo-embolism; AMI, acute myocardial infarction; ICU, intensive care unit; Hp, hypertension; DM, diabetes mellitus; arrh, arrhythmia; dec, deceased; sur, survivors.

## Data Availability

Not applicable.

## References

[1] Li W., Shi Z., Yu M., Ren W., Smith C., Epstein J.H., Wang H., Crameri G., Hu Z., Zhang H. (2005). Bats are natural reservoirs of SARS-like coronaviruses. Science.

[2] Ge X.Y., Li J.L., Yang X.L., Chmura A.A., Zhu G., Epstein J.H., Mazet J.K., Hu B., Zhang W., Peng C. (2013). Isolation and characterization of a bat SARS-like coronavirus that uses the ACE2 receptor. Nature.

[3] Yang L., Wu Z., Ren X., Yang F., He G., Zhang J., Dong J., Sun L., Zhu Y., Du J. (2013). Novel SARS-like betacoronaviruses in bats, China, 2011. Emerg. Infect. Dis..

[4] Hu B., Zeng L.P., Yang X.L., Ge X.Y., Zhang W., Li B., Xie J.Z., Shen X.R., Zhang Y.Z., Wang N. (2017). Discovery of a rich gene pool of bat SARS-related coronaviruses provides new insights into the origin of SARS coronavirus. PLoS Pathog..

[5] Menachery V.D., Yount B.L., Debbink K., Agnihothram S., Gralinski L.E., Plante J.A., Graham R.L., Scobey T., Ge X.Y., Donaldson E.F. (2015). A SARS-like cluster of circulating bat coronaviruses shows potential for human emergence. Nat. Med..

[6] Menachery V.D., Yount B.L., Sims A.C., Debbink K., Agnihothram S.S., Gralinski L.E., Graham R.L., Scobey T., Plante J.A., Royal S.R. (2016). SARS-like WIV1-CoV poised for human emergence. Proc. Natl. Acad. Sci. USA.

[7] Wang N., Li S.Y., Yang X.L., Huang H.M., Zhang Y.J., Guo H., Luo C.M., Miller M., Zhu G., Chmura A.A. (2018). Serological Evidence of Bat SARS-Related Coronavirus Infection in Humans, China. Virol. Sin..

[8] Zhou P., Yang X.L., Wang X.G., Hu B., Zhang L., Zhang W., Si H.R., Zhu Y., Li B., Huang C.L. (2020). A pneumonia outbreak associated with a new coronavirus of probable bat origin. Nature.

[9] WHO (2020). Director-General’s Opening Remarks at the Media Briefing on COVID-19-11 March 2020.

[10] Dong E., Du H., Gardner L. (2020). An interactive web-based dashboard to track COVID-19 in real time. Lancet Infect. Dis..

[11] Zhu N., Zhang D., Wang W., Li X., Yang B., Song J., Zhao X., Huang B., Shi W., Lu R. (2020). A Novel Coronavirus from Patients with Pneumonia in China, 2019. N. Engl. J. Med..

[12] Guo Y.R., Cao Q.D., Hong Z.S., Tan Y.Y., Chen S.D., Jin H.J., Tan K.S., Wang D.Y., Yan Y. (2020). The origin, transmission and clinical therapies on coronavirus disease 2019 (COVID-19) outbreak—An update on the status. Mil. Med. Res..

[13] Wan Y., Shang J., Graham R., Baric R.S., Li F. (2020). Receptor Recognition by the Novel Coronavirus from Wuhan: An Analysis Based on Decade-Long Structural Studies of SARS Coronavirus. J. Virol..

[14] Sheahan T., Rockx B., Donaldson E., Sims A., Pickles R., Corti D., Baric R. (2008). Mechanisms of zoonotic severe acute respiratory syndrome coronavirus host range expansion in human airway epithelium. J. Virol..

[15] Andersen K.G., Rambaut A., Lipkin W.I., Holmes E.C., Garry R.F. (2020). The proximal origin of SARS-CoV-2. Nat. Med..

[16] Bai Y., Yao L., Wei T., Tian F., Jin D.Y., Chen L., Wang M. (2020). Presumed Asymptomatic Carrier Transmission of COVID-19. JAMA.

[17] Kaźmierczak-Siedlecka K., Vitale E., Makarewicz W. (2020). COVID-19—Gastrointestinal and gut microbiota-related aspects. Eur. Rev. Med. Pharmacol. Sci..

[18] Aretz H.T. (1987). Myocarditis: The Dallas criteria. Hum. Pathol..

[19] Channappanavar R., Perlman S. (2017). Pathogenic human coronavirus infections: Causes and consequences of cytokine storm and immunopathology. Semin. Immunopathol..

[20] Huang C., Wang Y., Li X., Ren L., Zhao J., Hu Y., Zhang L., Fan G., Xu J., Gu X. (2020). Clinical features of patients infected with 2019 novel coronavirus in Wuhan, China. Lancet.

[21] Welt F., Shah P.B., Aronow H.D., Bortnick A.E., Henry T.D., Sherwood M.W., Young M.N., Davidson L.J., Kadavath S., Mahmud E. (2020). Catheterization Laboratory Considerations During the Coronavirus (COVID-19) Pandemic: From the ACC’s Interventional Council and SCAI. J. Am. Coll. Cardiol..

[22] Bansal M. (2020). Cardiovascular disease and COVID-19. Diabetes Metab. Syndr..

[23] Clerkin K.J., Fried J.A., Raikhelkar J., Sayer G., Griffin J.M., Masoumi A., Jain S.S., Burkhoff D., Kumaraiah D., Rabbani L. (2020). COVID-19 and Cardiovascular Disease. Circulation.

[24] Chan J.W., Ng C.K., Chan Y.H., Mok T.Y., Lee S., Chu S.Y., Law W.L., Lee M.P., Li P.C. (2003). Short term outcome and risk factors for adverse clinical outcomes in adults with severe acute respiratory syndrome (SARS). Thorax.

[25] Booth C.M., Matukas L.M., Tomlinson G.A., Rachlis A.R., Rose D.B., Dwosh H.A., Walmsley S.L., Mazzulli T., Avendano M., Derkach P. (2003). Clinical features and short-term outcomes of 144 patients with SARS in the greater Toronto area. JAMA.

[26] Badawi A., Ryoo S.G. (2016). Prevalence of comorbidities in the Middle East respiratory syndrome coronavirus (MERS-CoV): A systematic review and meta-analysis. Int. J. Infect. Dis..

[27] Du R.H., Liang L.R., Yang C.Q., Wang W., Cao T.Z., Li M., Guo G.Y., Du J., Zheng C.L., Zhu Q. (2020). Predictors of mortality for patients with COVID-19 pneumonia caused by SARS-CoV-2: A prospective cohort study. Eur. Respir. J..

[28] Zheng Y.Y., Ma Y.T., Zhang J.Y., Xie X. (2020). COVID-19 and the cardiovascular system. Nat. Rev. Cardiol..

[29] Zhou F., Yu T., Du R., Fan G., Liu Y., Liu Z., Xiang J., Wang Y., Song B., Gu X. (2020). Clinical course and risk factors for mortality of adult inpatients with COVID-19 in Wuhan, China: A retrospective cohort study. Lancet.

[30] Du Y., Tu L., Zhu P., Mu M., Wang R., Yang P., Wang X., Hu C., Ping R., Hu P. (2020). Clinical Features of 85 Fatal Cases of COVID-19 from Wuhan. A Retrospective Observational Study. Am. J. Respir. Crit. Care Med..

[31] Wang L., He W., Yu X., Hu D., Bao M., Liu H., Zhou J., Jiang H. (2020). Coronavirus disease 2019 in elderly patients: Characteristics and prognostic factors based on 4-week follow-up. J. Infect..

[32] Feng Y., Ling Y., Bai T., Xie Y., Huang J., Li J., Xiong W., Yang D., Chen R., Lu F. (2020). COVID-19 with Different Severities: A Multicenter Study of Clinical Features. Am. J. Respir. Crit. Care Med..

[33] Li M., Dong Y., Wang H., Guo W., Zhou H., Zhang Z., Tian C., Du K., Zhu R., Wang L. (2020). Cardiovascular disease potentially contributes to the progression and poor prognosis of COVID-19. Nutr. Metab. Cardiovasc. Dis..

[34] Yan Y., Yang Y., Wang F., Ren H., Zhang S., Shi X., Yu X., Dong K. (2020). Clinical characteristics and outcomes of patients with severe covid-19 with diabetes. BMJ Open Diabetes Res. Care.

[35] Li R., Tian J., Yang F., Lv L., Yu J., Sun G., Ma Y., Yang X., Ding J. (2020). Clinical characteristics of 225 patients with COVID-19 in a tertiary Hospital near Wuhan, China. J. Clin. Virol..

[36] Liang W.H., Guan W.J., Li C.C., Li Y.M., Liang H.R., Zhao Y., Liu X.Q., Sang L., Chen R.C., Tang C.L. (2020). Clinical characteristics and outcomes of hospitalised patients with COVID-19 treated in Hubei (epicentre) and outside Hubei (non-epicentre): A nationwide analysis of China. Eur. Respir. J..

[37] Gao M., Yang L., Chen X., Deng Y., Yang S., Xu H., Chen Z., Gao X. (2020). A study on infectivity of asymptomatic SARS-CoV-2 carriers. Respir Med..

[38] Guo T., Fan Y., Chen M., Wu X., Zhang L., He T., Wang H., Wan J., Wang X., Lu Z. (2020). Cardiovascular Implications of Fatal Outcomes of Patients with Coronavirus Disease 2019 (COVID-19). JAMA Cardiol..

[39] Deng Q., Hu B., Zhang Y., Wang H., Zhou X., Hu W., Cheng Y., Yan J., Ping H., Zhou Q. (2020). Suspected myocardial injury in patients with COVID-19: Evidence from front-line clinical observation in Wuhan, China. Int. J. Cardiol..

[40] Itelman E., Wasserstrum Y., Segev A., Avaky C., Negru L., Cohen D., Turpashvili N., Anani S., Zilber E., Lasman N. (2020). Clinical Characterization of 162 COVID-19 patients in Israel: Preliminary Report from a Large Tertiary Center. Isr. Med. Assoc. J..

[41] Gao L., Jiang D., Wen X.S., Cheng X.C., Sun M., He B., You L.N., Lei P., Tan X.W., Qin S. (2020). Prognostic value of NT-proBNP in patients with severe COVID-19. Respir. Res..

[42] Dong N., Cai J., Zhou Y., Liu J., Li F. (2020). End-Stage Heart Failure With COVID-19: Strong Evidence of Myocardial Injury by 2019-nCoV. JACC Heart Fail..

[43] Menter T., Haslbauer J.D., Nienhold R., Savic S., Hopfer H., Deigendesch N., Frank S., Turek D., Willi N., Pargger H. (2020). Postmortem examination of COVID-19 patients reveals diffuse alveolar damage with severe capillary congestion and variegated findings in lungs and other organs suggesting vascular dysfunction. Histopathology.

[44] Qin C., Zhou L., Hu Z., Yang S., Zhang S., Chen M., Yu H., Tian D.S., Wang W. (2020). Clinical Characteristics and Outcomes of COVID-19 Patients with a History of Stroke in Wuhan, China. Stroke.

[45] Zhang J., Lu S., Wang X., Jia X., Li J., Lei H., Liu Z., Liao F., Ji M., Lv X. (2020). Do underlying cardiovascular diseases have any impact on hospitalised patients with COVID-19?. Heart.

[46] Gao C., Cai Y., Zhang K., Zhou L., Zhang Y., Zhang X., Li Q., Li W., Yang S., Zhao X. (2020). Association of hypertension and antihypertensive treatment with COVID-19 mortality: A retrospective observational study. Eur. Heart J..

[47] Shao F., Xu S., Ma X., Xu Z., Lyu J., Ng M., Cui H., Yu C., Zhang Q., Sun P. (2020). In-hospital cardiac arrest outcomes among patients with COVID-19 pneumonia in Wuhan, China. Resuscitation.

[48] Pan F., Yang L., Li Y., Liang B., Li L., Ye T., Li L., Liu D., Gui S., Hu Y. (2020). Factors associated with death outcome in patients with severe coronavirus disease-19 (COVID-19): A case-control study. Int. J. Med. Sci..

[49] Chen Q., Xu L., Dai Y., Ling Y., Mao J., Qian J., Zhu W., Di W., Ge J. (2020). Cardiovascular manifestations in severe and critical patients with COVID-19. Clin. Cardiol..

[50] Xie J., Covassin N., Fan Z., Singh P., Gao W., Li G., Kara T., Somers V.K. (2020). Association Between Hypoxemia and Mortality in Patients With COVID-19. Mayo Clin. Proc..

[51] Shi S., Qin M., Shen B., Cai Y., Liu T., Yang F., Gong W., Liu X., Liang J., Zhao Q. (2020). Association of Cardiac Injury with Mortality in Hospitalized Patients With COVID-19 in Wuhan, China. JAMA Cardiol..

[52] Li P., Chen L., Liu Z., Pan J., Zhou D., Wang H., Gong H., Fu Z., Song Q., Min Q. (2020). Clinical features and short-term outcomes of elderly patients with COVID-19. Int. J. Infect. Dis..

[53] Edler C., Schröder A.S., Aepfelbacher M., Fitzek A., Heinemann A., Heinrich F., Klein A., Langenwalder F., Lütgehetmann M., Meißner K. (2020). Dying with SARS-CoV-2 infection—An autopsy study of the first consecutive 80 cases in Hamburg, Germany. Int. J. Legal. Med..

[54] Creel-Bulos C., Hockstein M., Amin N., Melhem S., Truong A., Sharifpour M. (2020). Acute Cor Pulmonale in Critically Ill Patients with Covid-19. N. Engl. J. Med..

[55] Ferguson J., Rosser J.I., Quintero O., Scott J., Subramanian A., Gumma M., Rogers A., Kappagoda S. (2020). Characteristics and Outcomes of Coronavirus Disease Patients under Nonsurge Conditions, Northern California, USA, March–April 2020. Emerg. Infect. Dis..

[56] Yang L., Liu J., Zhang R., Li M., Li Z., Zhou X., Hu C., Tian F., Zhou F., Lei Y. (2020). Epidemiological and clinical features of 200 hospitalized patients with corona virus disease 2019 outside Wuhan, China: A descriptive study. J. Clin. Virol..

[57] Ren Z.L., Hu R., Wang Z.W., Zhang M., Ruan Y.L., Wu Z.Y., Wu H.B., Hu X.P., Hu Z.P., Ren W. (2020). Epidemiologic and clinical characteristics of heart transplant recipients during the 2019 coronavirus outbreak in Wuhan, China: A descriptive survey report. J. Heart Lung Transpl..

[58] Zhao M., Wang M., Zhang J., Gu J., Zhang P., Xu Y., Ye J., Wang Z., Ye D., Pan W. (2020). Comparison of clinical characteristics and outcomes of patients with coronavirus disease 2019 at different ages. Aging.

[59] Goicoechea M., Sánchez Cámara L.A., Macías N., Muñoz de Morales A., Rojas Á.G., Bascuñana A., Arroyo D., Vega A., Abad S., Verde E. (2020). COVID-19: Clinical course and outcomes of 36 hemodialysis patients in Spain. Kidney Int..

[60] Lodigiani C., Iapichino G., Carenzo L., Cecconi M., Ferrazzi P., Sebastian T., Kucher N., Studt J.D., Sacco C., Alexia B. (2020). Venous and arterial thromboembolic complications in COVID-19 patients admitted to an academic hospital in Milan, Italy. Thromb. Res..

[61] Xie Y., You Q., Wu C., Cao S., Qu G., Yan X., Han X., Wang C., Zhang H. (2020). Impact of Cardiovascular Disease on Clinical Characteristics and Outcomes of Coronavirus Disease 2019 (COVID-19). Circ. J..

[62] Buckner F.S., McCulloch D.J., Atluri V., Blain M., McGuffin S.A., Nalla A.K., Huang M.L., Greninger A.L., Jerome K.R., Cohen S.A. (2020). Clinical Features and Outcomes of 105 Hospitalized patients with COVID-19 in Seattle, Washington. Clin. Infect. Dis..

[63] Zheng Y., Sun L.J., Xu M., Pan J., Zhang Y.T., Fang X.L., Fang Q., Cai H.L. (2020). Clinical characteristics of 34 COVID-19 patients admitted to intensive care unit in Hangzhou, China. J. Zhejiang Univ. Sci. B.

[64] Wan S., Xiang Y., Fang W., Zheng Y., Li B., Hu Y., Lang C., Huang D., Sun Q., Xiong Y. (2020). Clinical features and treatment of COVID-19 patients in northeast Chongqing. J. Med. Virol..

[65] Rath D., Petersen-Uribe Á., Avdiu A., Witzel K., Jaeger P., Zdanyte M., Heinzmann D., Tavlaki E., Müller K., Gawaz M.P. (2020). Impaired cardiac function is associated with mortality in patients with acute COVID-19 infection. Clin. Res. Cardiol..

[66] Biagi A., Rossi L., Malagoli A., Zanni A., Sticozzi C., Comastri G., Gandolfi S., Villani G.Q. (2020). Clinical and epidemiological characteristics of 320 deceased patients with COVID-19 in an Italian Province: A retrospective observational study. J. Med. Virol..

[67] Sabatino J., Ferrero P., Chessa M., Bianco F., Ciliberti P., Secinaro A., Oreto L., Avesani M., Bucciarelli V., Calcaterra G. (2020). COVID-19 and Congenital Heart Disease: Results from a Nationwide Survey. J. Clin. Med..

[68] Shi Q., Zhang X., Jiang F., Zhang X., Hu N., Bimu C., Feng J., Yan S., Guan Y., Xu D. (2020). Clinical Characteristics and Risk Factors for Mortality of COVID-19 Patients with Diabetes in Wuhan, China: A Two-Center, Retrospective Study. Diabetes Care.

[69] Galloway J.B., Norton S., Barker R.D., Brookes A., Carey I., Clarke B.D., Jina R., Reid C., Russell M.D., Sneep R. (2020). A clinical risk score to identify patients with COVID-19 at high risk of critical care admission or death: An observational cohort study. J. Infect..

[70] Palmieri L., Vanacore N., Donfrancesco C., Lo Noce C., Canevelli M., Punzo O., Raparelli V., Pezzotti P., Riccardo F., Bella A. (2020). Clinical Characteristics of Hospitalized Individuals Dying with COVID-19 by Age Group in Italy. J. Gerontol A Biol. Sci. Med. Sci..

[71] Wei J.F., Huang F.Y., Xiong T.Y., Liu Q., Chen H., Wang H., Huang H., Luo Y.C., Zhou X., Liu Z.Y. (2020). Acute myocardial injury is common in patients with COVID-19 and impairs their prognosis. Heart.

[72] Jain S., Workman V., Ganeshan R., Obasare E.R., Burr A., DeBiasi R.M., Freeman J.V., Akar J., Lampert R., Rosenfeld L.E. (2020). Enhanced electrocardiographic monitoring of patients with Coronavirus Disease 2019. Heart Rhythm..

[73] Inciardi R.M., Lupi L., Zaccone G., Italia L., Raffo M., Tomasoni D., Cani D.S., Cerini M., Farina D., Gavazzi E. (2020). Cardiac Involvement in a Patient with Coronavirus Disease 2019 (COVID-19). JAMA Cardiol..

[74] Chen T., Wu D., Chen H., Yan W., Yang D., Chen G., Ma K., Xu D., Yu H., Wang H. (2020). Clinical characteristics of 113 deceased patients with coronavirus disease 2019: Retrospective study. BMJ.

[75] Oudit G.Y., Kassiri Z., Jiang C., Liu P.P., Poutanen S.M., Penninger J.M., Butany J. (2009). SARS-coronavirus modulation of myocardial ACE2 expression and inflammation in patients with SARS. Eur. J. Clin. Investig..

[76] Peiris J.S., Chu C.M., Cheng V.C., Chan K.S., Hung I.F., Poon L.L., Law K.I., Tang B.S., Hon T.Y., Chan C.S. (2003). Clinical progression and viral load in a community outbreak of coronavirus-associated SARS pneumonia: A prospective study. Lancet.

[77] Akhmerov A., Marbán E. (2020). COVID-19 and the Heart. Circ. Res..

[78] Yao X.H., Li T.Y., He Z.C., Ping Y.F., Liu H.W., Yu S.C., Mou H.M., Wang L.H., Zhang H.R., Fu W.J. (2020). A pathological report of three COVID-19 cases by minimal invasive autopsies. Zhonghua Bing Li Xue Za Zhi.

[79] Mehta P., McAuley D.F., Brown M., Sanchez E., Tattersall R.S., Manson J.J., HLH Across Speciality Collaboration, UK (2020). COVID-19: Consider cytokine storm syndromes and immunosuppression. Lancet.

[80] Siddiqi H.K., Mehra M.R. (2020). COVID-19 illness in native and immunosuppressed states: A clinical-therapeutic staging proposal. J. Heart Lung Transpl..

[81] Ruan Q., Yang K., Wang W., Jiang L., Song J. (2020). Clinical predictors of mortality due to COVID-19 based on an analysis of data of 150 patients from Wuhan, China. Intensive Care Med..

[82] Wang D., Hu B., Hu C., Zhu F., Liu X., Zhang J., Wang B., Xiang H., Cheng Z., Xiong Y. (2020). Clinical Characteristics of 138 Hospitalized Patients With 2019 Novel Coronavirus-Infected Pneumonia in Wuhan, China. JAMA.

[83] Ma K.L., Liu Z.H., Cao C.F., Liu M.K., Liao J., Zou J.B., Kong L.X., Wan K.Q., Zhang J., Wang Q.B. COVID-19 myocarditis and severity factors: An adult cohort study. MedRxiv.

[84] Xu Z., Shi L., Wang Y., Zhang J., Huang L., Zhang C., Liu S., Zhao P., Liu H., Zhu L. (2020). Pathological findings of COVID-19 associated with acute respiratory distress syndrome. Lancet Respir. Med..

[85] Fung G., Luo H., Qiu Y., Yang D., McManus B. (2016). Myocarditis. Circ. Res..

[86] Chen L., Li X., Chen M., Feng Y., Xiong C. (2020). The ACE2 expression in human heart indicates new potential mechanism of heart injury among patients infected with SARS-CoV-2. Cardiovasc. Res..

[87] Hu H., Ma F., Wei X., Fang Y. (2020). Coronavirus fulminant myocarditis saved with glucocorticoid and human immunoglobulin. Eur. Heart J..

[88] Ammirati E., Cipriani M., Moro C., Raineri C., Pini D., Sormani P., Mantovani R., Varrenti M., Pedrotti P., Conca C. (2018). Registro Lombardo delle Miocarditi. Clinical Presentation and Outcome in a Contemporary Cohort of Patients with Acute Myocarditis: Multicenter Lombardy Registry. Circulation.

[89] Liu Y., Yang Y., Zhang C., Huang F., Wang F., Yuan J., Wang Z., Li J., Li J., Feng C. (2020). Clinical and biochemical indexes from 2019-nCoV infected patients linked to viral loads and lung injury. Sci. China Life Sci..

[90] American College of Cardiology ACC-CCA Webinar: COVID-19 Severe Case Management. Accessed March 27, **2020**.

[91] Corrales-Medina V.F., Alvarez K.N., Weissfeld L.A., Angus D.C., Chirinos J.A., Chang C.C., Newman A., Loehr L., Folsom A.R., Elkind M.S. (2015). Association between hospitalization for pneumonia and subsequent risk of cardiovascular disease. JAMA.

[92] Kwong J.C., Schwartz K.L., Campitelli M.A., Chung H., Crowcroft N.S., Karnauchow T., Katz K., Ko D.T., McGeer A.J., McNally D. (2018). Acute myocardial infarction after laboratory-confirmed influenza infection. N. Engl. J. Med..

[93] Long B., Brady W.J., Koyfman A., Gottlieb M. (2020). Cardiovascular complications in COVID-19. Am. J. Emerg. Med..

[94] Liu K., Fang Y.Y., Deng Y., Liu W., Wang M.F., Ma J.P., Xiao W., Wang Y.N., Zhong M.H., Li C.H. (2020). Clinical characteristics of novel coronavirus cases in tertiary hospitals in Hubei Province. Chin. Med. J..

[95] Yang X., Yu Y., Xu J., Shu H., Xia J., Liu H., Wu Y., Zhang L., Yu Z., Fang M. (2020). Clinical course and outcomes of critically ill patients with SARS-CoV-2 pneumonia in Wuhan, China: A single-centered, retrospective, observational study. Lancet Respir. Med..

[96] Chen C., Zhou Y., Wang D.W. (2020). SARS-CoV-2: A potential novel etiology of fulminant myocarditis. Herz.

[97] Buzon J., Roignot O., Lemoine S., Perez P., Kimmoun A., Levy B., Novy E. (2015). Takotsubo Cardiomyopathy Triggered by Influenza A Virus. Intern. Med..

[98] Zompatori M., Ciccarese F., Fasano L. (2014). Overview of current lung imaging in acute respiratory distress syndrome. Eur. Respir Rev..

[99] Ferguson N.D., Fan E., Camporota L., Antonelli M., Anzueto A., Beale R., Brochard L., Brower R., Esteban A., Gattinoni L. (2012). The Berlin definition of ARDS: An expanded rationale, justification, and supplementary material. Intensive Care Med..

[100] Ranieri V.M., Rubenfeld G.D., Thompson B.T., Ferguson N.D., Caldwell E., Fan E., Camporota L., Slutsky A.S., ARDS Definition Task Force (2012). Acute respiratory distress syndrome: The Berlin Definition. JAMA.

[101] Karmpaliotis D., Kirtane A.J., Ruisi C.P., Polonsky T., Malhotra A., Talmor D., Kosmidou I., Jarolim P., de Lemos J.A., Sabatine M.S. (2007). Diagnostic and prognostic utility of brain natriuretic Peptide in subjects admitted to the ICU with hypoxic respiratory failure due to noncardiogenic and cardiogenic pulmonary edema. Chest.

[102] MacLaren G., Fisher D., Brodie D. (2020). Preparing for the Most Critically Ill Patients With COVID-19: The Potential Role of Extracorporeal Membrane Oxygenation. JAMA.

[103] Xie Y., Wang X., Yang P., Zhang S. (2020). COVID-19 Complicated by Acute Pulmonary Embolism. Radiol. Cardiothorac. Imaging.

[104] Danzi G.B., Loffi M., Galeazzi G., Gherbesi E. (2020). Acute pulmonary embolism and COVID-19 pneumonia: A random association?. Eur. Heart J..

[105] Tang N., Li D., Wang X., Sun Z. (2020). Abnormal coagulation parameters are associated with poor prognosis in patients with novel coronavirus pneumonia. J. Thromb. Haemost..

[106] Chen J., Wang X., Zhang S., Lin B., Wu X., Wang Y., Wang X., Yang M., Sun J., Xie Y. (2020). Characteristics of Acute Pulmonary Embolism in Patients With COVID-19 Associated Pneumonia from the City of Wuhan. Clin. Appl. Thromb. Hemost..

[107] Tang N., Bai H., Chen X., Gong J., Li D., Sun Z. (2020). Anticoagulant treatment is associated with decreased mortality in severe coronavirus disease 2019 patients with coagulopathy. J. Thromb. Haemost..

[108] Fox S.E., Akmatbekov A., Harbert J.L., Li G., Quincy Brown J., Vander Heide R.S. (2020). Pulmonary and cardiac pathology in African American patients with COVID-19: An autopsy series from New Orleans. Lancet Respir. Med..

[109] Du L., Kao R.Y., Zhou Y., He Y., Zhao G., Wong C., Jiang S., Yuen K.Y., Jin D.Y., Zheng B.J. (2020). Cleavage of spike protein of SARS coronavirus by protease factor Xa is associated with viral infectivity. Biochem. Biophys. Res. Commun..

[110] Li F., Cai J., Dong N. (2020). First cases of COVID-19 in heart transplantation from China. J. Heart Lung Transpl..

[111] Aslam S., Mehra M.R. (2020). COVID-19: Yet another coronavirus challenge in transplantation. J. Heart Lung Transpl..

[112] Wu Q., Zhou L., Sun X., Yan Z., Hu C., Wu J., Xu L., Li X., Liu H., Yin P. (2017). Altered Lipid Metabolism in Recovered SARS Patients Twelve Years after Infection. Sci. Rep..

[113] Wong K.T., Antonio G.E., Hui D.S., Lee N., Yuen E.H., Wu A., Leung C.B., Rainer T.H., Cameron P., Chung S.S. (2003). Thin-section CT of severe acute respiratory syndrome: Evaluation of 73 patients exposed to or with the disease. Radiology.

[114] Cangemi R., Calvieri C., Falcone M., Bucci T., Bertazzoni G., Scarpellini M.G., Barillà F., Taliani G., Violi F., SIXTUS Study Group (2015). Relation of Cardiac Complications in the Early Phase of Community-Acquired Pneumonia to Long-Term Mortality and Cardiovascular Events. Am. J. Cardiol..

[115] Wang Y., Dong C., Hu Y., Li C., Ren Q., Zhang X., Shi H., Zhou M. (2020). Temporal Changes of CT Findings in 90 Patients with COVID-19 Pneumonia: A Longitudinal Study. Radiology.

[116] Meng H., Xiong R., He R., Lin W., Hao B., Zhang L., Lu Z., Shen X., Fan T., Jiang W. (2020). CT imaging and clinical course of asymptomatic cases with COVID-19 pneumonia at admission in Wuhan, China. J. Infect..

[117] Hosseiny M., Kooraki S., Gholamrezanezhad A., Reddy S., Myers L. (2020). Radiology Perspective of Coronavirus Disease 2019 (COVID-19): Lessons from Severe Acute Respiratory Syndrome and Middle East Respiratory Syndrome. AJR Am. J. Roentgenol..

[118] Zhang H., Penninger J.M., Li Y., Zhong N., Slutsky A.S. (2020). Angiotensin-converting enzyme 2 (ACE2) as a SARS-CoV-2 receptor: Molecular mechanisms and potential therapeutic target. Intensive Care Med..

[119] Becker R.C. (2020). COVID-19-associated vasculitis and vasculopathy. J. Thromb. Thrombolysis.

[120] Shah R.M., Shah M., Shah S., Li A., Jauhar S. (2021). Takotsubo Syndrome and COVID-19: Associations and Implications. Curr. Probl. Cardiol..

[121] Cannizzaro E., Cannizzaro C., Martorana D., Moscadini S., Lo Coco D. (2012). Effects of shift work on cardiovascular activity, serum cortisol and white blood cell count in a group of Italian fishermen. Euro Mediterr. Biomed. J..

[122] Hollander J.E., Carr B.G. (2020). Virtually Perfect? Telemedicine for Covid-19. N. Engl. J. Med..

